# BcWRKY25-BcWRKY33A-BcLRP1/BcCOW1 module promotes root development for improved salt tolerance in Bok choy

**DOI:** 10.1093/hr/uhae280

**Published:** 2024-09-28

**Authors:** Huiyu Wang, Yushan Zheng, Meiyun Wang, Wusheng Liu, Ying Li, Dong Xiao, Tongkun Liu, Xilin Hou

**Affiliations:** State Key Laboratory of Crop Genetics & Germplasm Enhancement, Key Laboratory of Biology and Genetic Improvement of Horticultural Crops (East China), Ministry of Agriculture and Rural Affairs of China, Engineering Research Center of Germplasm Enhancement and Utilization of Horticultural Crops, Ministry of Education of China, Nanjing Agricultural University, No.1 Weigang Road, Xuanwu District, Nanjing 210095, China; Department of Plant Science and Technology, Beijing Key Laboratory of New Technology in Agricultural Application, National Demonstration Center for Experimental Plant Production Education, Beijing University of Agriculture, No.7 Beinong Road, Changping District, Beijing 102206, China; State Key Laboratory of Crop Genetics & Germplasm Enhancement, Key Laboratory of Biology and Genetic Improvement of Horticultural Crops (East China), Ministry of Agriculture and Rural Affairs of China, Engineering Research Center of Germplasm Enhancement and Utilization of Horticultural Crops, Ministry of Education of China, Nanjing Agricultural University, No.1 Weigang Road, Xuanwu District, Nanjing 210095, China; State Key Laboratory of Crop Genetics & Germplasm Enhancement, Key Laboratory of Biology and Genetic Improvement of Horticultural Crops (East China), Ministry of Agriculture and Rural Affairs of China, Engineering Research Center of Germplasm Enhancement and Utilization of Horticultural Crops, Ministry of Education of China, Nanjing Agricultural University, No.1 Weigang Road, Xuanwu District, Nanjing 210095, China; Department of Horticultural Science, North Carolina State University, 2101 Hillsborough Street, Raleigh, North Carolina 27607, USA; State Key Laboratory of Crop Genetics & Germplasm Enhancement, Key Laboratory of Biology and Genetic Improvement of Horticultural Crops (East China), Ministry of Agriculture and Rural Affairs of China, Engineering Research Center of Germplasm Enhancement and Utilization of Horticultural Crops, Ministry of Education of China, Nanjing Agricultural University, No.1 Weigang Road, Xuanwu District, Nanjing 210095, China; Nanjing Suman Plasma Engineering Research Institute, Nanjing Agricultural University, No.7 Yinian Road, Jiangning District, Nanjing 210095, China; State Key Laboratory of Crop Genetics & Germplasm Enhancement, Key Laboratory of Biology and Genetic Improvement of Horticultural Crops (East China), Ministry of Agriculture and Rural Affairs of China, Engineering Research Center of Germplasm Enhancement and Utilization of Horticultural Crops, Ministry of Education of China, Nanjing Agricultural University, No.1 Weigang Road, Xuanwu District, Nanjing 210095, China; State Key Laboratory of Crop Genetics & Germplasm Enhancement, Key Laboratory of Biology and Genetic Improvement of Horticultural Crops (East China), Ministry of Agriculture and Rural Affairs of China, Engineering Research Center of Germplasm Enhancement and Utilization of Horticultural Crops, Ministry of Education of China, Nanjing Agricultural University, No.1 Weigang Road, Xuanwu District, Nanjing 210095, China; State Key Laboratory of Crop Genetics & Germplasm Enhancement, Key Laboratory of Biology and Genetic Improvement of Horticultural Crops (East China), Ministry of Agriculture and Rural Affairs of China, Engineering Research Center of Germplasm Enhancement and Utilization of Horticultural Crops, Ministry of Education of China, Nanjing Agricultural University, No.1 Weigang Road, Xuanwu District, Nanjing 210095, China; Nanjing Suman Plasma Engineering Research Institute, Nanjing Agricultural University, No.7 Yinian Road, Jiangning District, Nanjing 210095, China

## Abstract

Root development is a complex process involving phytohormones and transcription factors. Our previous research has demonstrated that *BcWRKY33A* is significantly expressed in Bok choy roots under salt stress, and heterologous expression of *BcWRKY33A* increases salt tolerance and promotes root development in transgenic *Arabidopsis*. However, the precise molecular mechanisms by which BcWRKY33A governs root development remain elusive. Here, we investigated the role of *BcWRKY33A* in both root elongation and root hair formation in transgenic Bok choy roots. Our data indicated that overexpression of *BcWRKY33A* stimulated root growth and stabilized root hair morphology, while silencing *BcWRKY33A* prevented primary root elongation and resulted in abnormal root hairs morphology. Meanwhile, our research uncovered that BcWRKY33A directly binds to the promoters of *BcLRP1* and *BcCOW1*, leading to an upregulation of their expression. In transgenic Bok choy roots, increased *BcLRP1* and *BcCOW1* transcript levels improved primary root elongation and root hair formation, respectively. Additionally, we pinpointed *BcWRKY25* as a NaCl-responsive gene that directly stimulates the expression of *BcWRKY33A* in response to salt stress. All results shed light on the regulatory mechanisms governing root development by BcWRKY25-BcWRKY33A-BcLRP1/BcCOW1 module and propose potential strategies for improving salt tolerance in Bok choy.

## Introduction

The root is the underground part of the plant and is mainly responsible for stabilizing and supporting the development of the aboveground parts of the plant, storing and transporting water and nutrients, and interacting with the living organisms in the soil to ensure the survival and health of the plant [1, 2]. The plasticity of the plant root system is influenced by endogenous genetic elements and exogenous environmental variables [3]. In seed plants, the primary roots develop from the radicle. Thin and individual epidermal cells, also known as root hairs, spread out from the primary roots to increase their surface area [4]. The primary roots also develop lateral roots, which have the same internal structure as the primary roots. As the plant grows, the architecture of the root system is constantly modified to meet the growth needs of the plant by dynamically adjusting the growth and development, density, and morphology of the primary, lateral roots, and root hairs [5, 6].

Considering the importance of robust root systems for overall plant growth, extensive research efforts have been dedicated to elucidating the genetic regulators that shape and control root architecture and development. For example, IAA18 interacts with the transcriptional activators AUXIN RESPONSE FACTOR 7 (ARF7) and ARF19 to promote lateral root formation. A gain-of-function mutation in *IAA18* domain II in *Arabidopsis* caused the *crane* mutant phenotype with a significantly reduced lateral root system [7]. Similarly, mutation in *IAA28* suppressed the elongation of lateral roots [8]. In addition, *SUPERCENTIPEDE1* (*SCN1*) regulates the position and morphology of root hairs [9]. The *scn1* mutant formed multiple root hairs from a single initiation site, the root hairs were substantial outgrowths with numerous irregular bulges, or the hairs formed at ectopic sites on the trichoblast surface. The ethylene-activated transcription factor ETHYLENE-INSENSITIVE 3 (EIN3) physically interacts with ROOT HAIR DEFECTIVE 6 (RHD6), a known positive regulator of hair cells, and directly co-activates the hair length-determining gene *RHD6-LIKE 4* (*RSL4*) to promote root hair elongation [10]. The trihelix transcription factor GT-2-LIKE1 (GTL1) and its homolog DF1 can bind directly to the *RSL4* promoter and regulate its expression to suppress root hair growth [11]. *TIP GROWTH DEFECTIVE1* (*TIP1*) plays a pivotal role in plant cellular expansion, and mutations in this gene are associated with impaired growth not only in root hair development but also in pollen tube elongation in *Arabidopsis* [12].

WRKY transcription factors constitute a prominent class of sequence-specific DNA-binding proteins, predominantly identified in eukaryotes and highly prevalent in the plant kingdom, where they play crucial roles in gene regulation [13], and are a crucial group of regulators of root development [14]. As an auxin-inducible gene in *Arabidopsis*, *WRKY23* could stimulate local flavanol synthesis and maintaining cell identity in the primary root meristem, which is then involved in root growth [15]. The wheat *TaWRKY51* RNAi lines and mutants had fewer lateral roots, while the *TaWRKY51* overexpression lines showed the opposite phenotype [16]. In rice, it has been reported that both the common tuber leaf fungus *Magnaporthe grisea* and auxin can induce the expression of *OsWRKY31*. Transgenic rice plants overexpressing *OsWRKY31* showed greater resistance to *M. grisea* and impaired lateral roots [17]. This regulation was mediated by the rice genes *INDOLE-3-ACETIC ACID INDUCIBLE 4* (*OsIAA4*) and *CROWN ROOTLESS 1* (*OsCrl1*), which encode negative regulators of auxin signaling [17]. With respect to root hair development, WRKY75 has been shown to suppress root hair formation in *Arabidopsis* through direct modulation of *CAPRICE* gene expression [14].

In addition, WRKY33 is pivotal in mediating both tolerance to abiotic and biotic stresses [13]. Arabidopsis *AtWRKY33* is renowned for conferring resistance to necrotrophic fungi and is integral to the plant’s mechanism for responding to salt stress [18–21]. For example, *AtWRKY33*, an upstream regulator of *AtCYP94B1*, ensures the formation of an apoplastic barrier in the root [21]. *AtWRKY33* acts as a negative regulator mediating phosphate starvation-induced root architecture remodeling [22]. Our previous research found that there were three copies of WRKY33 in Bok choy, all of which were induced by salt stress, with *BcWRKY33A* (*BraC04g029190.1*) being the most markedly induced and accumulated in the roots of Bok choy under salt stress, and overexpression of *BcWRKY33A* could increase the length of lateral and primary roots in transgenic *Arabidopsis* plants [23]. However, the molecular regulatory pathway of *BcWRKY33A* during root development is not yet fully understood.

Bok choy is widely grown in Asia due to its high yield and short growth cycle, but its shallow roots make it vulnerable to abiotic stressors such as drought and salinity [24]. Understanding the mechanism of root development of Bok choy will be helpful for breeding stress-resistant plant varieties. Here, we investigated the molecular mechanisms underlying BcWRKY33A-mediated root development in Bok choy. Finally, we established a novel regulatory network to reveal how *BcWRKY33A* regulates root development in response to salt stress in Boy choy.

## Results

### The expression of BcWRKY33A in Bok choy root was influenced by various abiotic stress factors

**Fig. 1:**
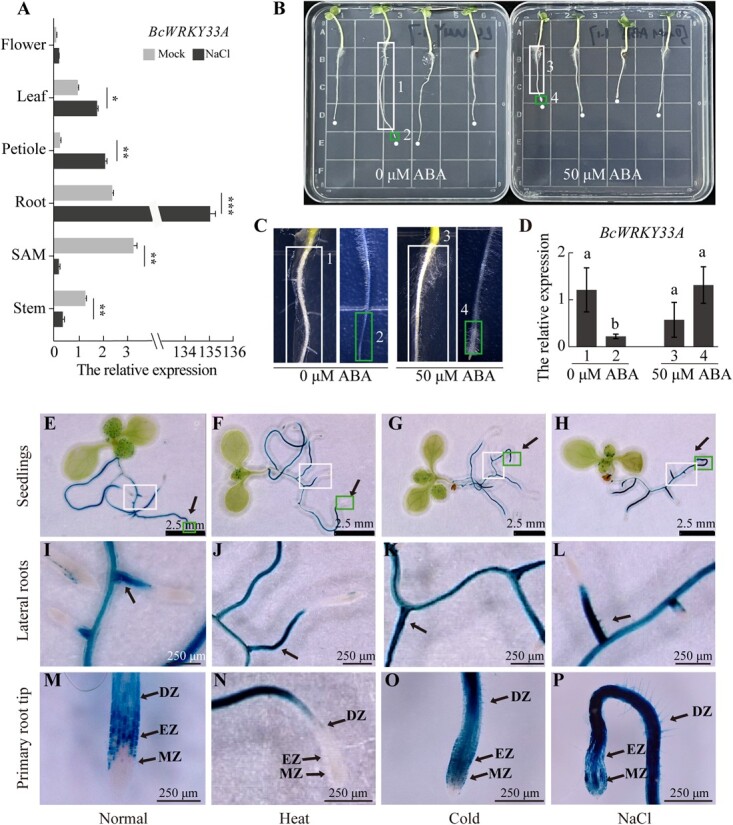
***BcWRKY33A* expression pattern in Bok choy and transgenic Arabidopsis under abiotic stress. (A**) The relative expression level of *BcWRKY33A* in different tissues of 1-month-old Bok choy under mock or NaCl treatment. Leaf (lamina), SAM (Shoot apical meristem). (**B**) Root morphology of 5-day-old Bok choy treated with 0 or 50 μM abscisic acid (ABA). Boxes 1 and 3 indicate DZ. Boxes 2 and 4 indicate root tips including the cap, MZ, and EZ. (**C**) The root enlarged image of Boxes 1–4 in Fig. 1b and 1c. (**D**) The relative expression level of *BcWRKY33A* in the different parts of roots (Boxes 1–4 in Fig. 1b and 1c). Different letters indicate statistically significant differences at the level of *P* < .05 (Student’s *t*-test). (**E–P**) GUS staining analysis of the seedlings **(E–H)**, lateral roots **(I–L)**, and primary root tips **(M–P)** of *BcWRKY33A:GUS* transgenic Arabidopsis under normal (23°C, 0 h), heat (43°C, 6 h), cold (4°C, 6 h), or NaCl (100 mM, 24 h) treatments. I–L was the lateral roots enlarged image of big white boxes in E–H, M–P was the primary roots enlarged image of small green boxes in E–H. DZ, differentiation zone; MZ, meristem zone; EZ, elongation zone. Error bars represent standard deviation (SD). The data are the mean ± SD of three biological replicates. ^*^P < 0.05, ^**^P < 0.01, ^***^P < 0.001 (Student’s *t*-test).

To elucidate the expression profiles of *BcWRKY33A* across various tissues of 1-month-old Bok choy inbred line ‘Suzhouqing’, quantitative real-time PCR (qRT-PCR) results showed *BcWRKY33A* was primarily expressed in the shoot apical meristem (SAM), root, stem, and leaf (lamina), but not in the flower or petiole (Fig. 1a). After 24 h of treatment with 150 mM NaCl, the relative expression of *BcWRKY33A* in roots increased ~53-fold compared to the mock treatment (Fig. 1a), which is consistent with previous study [23]. In addition, the expression of *BcWRKY33A* was increased 8-fold and 1.7-fold in the petiole and leaf of Bok choy, respectively, while it decreased 4-fold and 16-fold in the stem and shoot under 150 mM NaCl treatment, respectively (Fig. 1a).

 Abscisic acid (ABA) is an essential hormone for responding to diverse stress signals, and exogenous application of ABA may mimic the impact of abiotic stress conditions on plants [25]. We preliminarily explored the expression pattern of *BcWRKY33A* under ABA treatment. Under the mock treatment (0 μM ABA) for 24 h, 5-day-old ‘Suzhouqing’ seedlings formed lateral roots in the differentiation zone (Box 1 in Fig. 1b and 1c). However, under 50-μM ABA treatment for 24 h, both lateral root emergence and primary root elongation were inhibited, and many root hairs were formed in the differentiation zone (Box 3 and 4 in Fig. 1b and 1c). In addition, we found that the expression of *BcWRKY33A* in the differentiation zone (Box 1 and 3 in Fig. 1b) showed no significant difference between the mock and ABA treatments (Fig. 1d). However, it increased significantly at the root tips (Box 2 and 4 in Fig. 1b) after ABA treatment compared to mock treatment (Fig. 1d), which indicates the expression of *BcWRKY33A* in the primary root tips was induced by ABA.

Our findings indicated that the activity of the BcWRKY33A promoter is largely confined to the roots and leaf trichomes [26]. To investigate the activity of the *BcWRKY33A*’s promoter in root tissues, we generated *BcWRKY33A:GUS* transgenic lines and used histochemical β-glucuronidase staining (GUS) analysis (Fig. 1e–1p). The results showed that *BcWRKY33A* was constantly expressed at the base of lateral roots under normal, heat, cold, or NaCl conditions (Fig. 1i–1l). Under normal conditions, GUS signalling was mainly detected in the differentiation zones (DZ) and elongation zones (EZ) (Fig. 1m). Under heat stress, GUS signaling was significantly suppressed in the DZ and EZ (Fig. 1n). Interestingly, under cold or NaCl treatment, GUS signaling was significantly increased in the meristematic zones (MZ) and EZ (Fig. 1o–1p), which was consistent with our previous study [23]. All these data indicate that the expression of *BcWRKY33A* in Bok choy root was affected by various abiotic stress factors.

### Overexpression of *BcWRKY33A* promote root elongation and root hair formation in Bok choy

We first observed the root morphology among *BcWRKY33A* transgenic *Arabidopsis* lines (#1 and #3) and wild type (WT). Under normal conditions, the primary root of Line #1 was longer than that of WT (Fig. S1a–b). At 150 mM NaCl concentration, primary taproot and lateral roots of Line #1 and #3 were significantly longer than those of WT (Fig. S1a, c, d). Meanwhile, propidium iodide (PI) staining results showed Line #3 had more root hairs on the primary roots than WT (Fig. S1e–f).

**Fig. 2:**
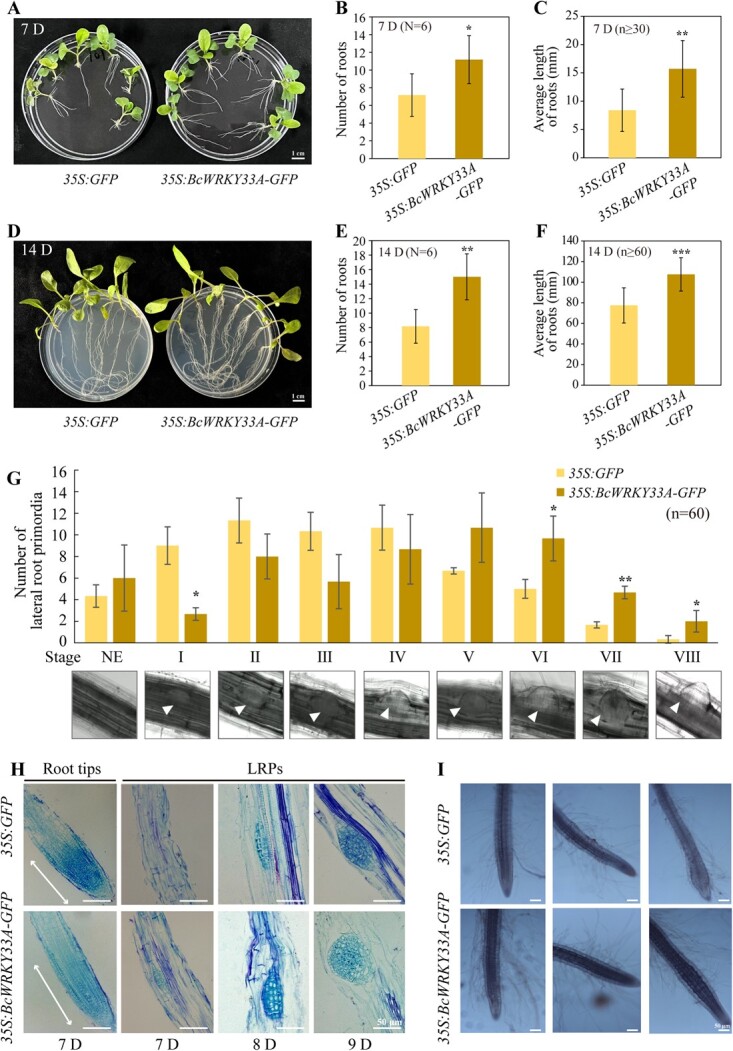
**Overexpression of *BcWRKY33A* in Bok choy roots promotes root elongation and root hair formation.** (**A–F**) The phenotype (**A, D**), roots number (**B, E**), average root lengths (**C, F**) of the 7- and 14-day (D)-old *35S:GFP* and *35S:BcWRKY33A-GFP* plants. Six seedlings per line were used. *N* represents number of seedlings, *n* represents number of roots. *35S:GFP* was used as the control. (**G**) The number of LRPs in the 8-day-old *35S:BcWRKY33A-GFP* and *35S:GFP* plants (the critical period for LRP emergence). *n* represents number of roots. (**H**) The root tips and LRP phenotype in the 7- to 9-day-old *35S:BcWRKY33A-GFP* and *35S:GFP* plants (at least 20 roots from six seedlings per line). (**I**) The root hairs in the 16-day-old *35S:BcWRKY33A-GFP* and *35S:GFP* plants (at least 20 roots from six seedlings per line). Error bars represent SD. The data are the mean ± SD of three biological replicates. ^*^P < 0.05, ^**^P < 0.01, ^***^P < 0.001 (Student’s *t*-test).

To better comprehend the function of *BcWRKY33A* in the Bok choy root, we generated *35S:BcWRKY33A-GFP* transgenic Bok choy by *Agrobacterium rhizogenes*-mediated transient transformation (Fig. S2) [27]. Compared with the control, 7- and 14-day-old 35S:BcWRKY33A-GFP roots exhibited significantly higher root number and average root length (Fig. 2a–2f). Following previous research, it has been identified that the development of lateral root primordia in hairy roots is a process that comprises eight distinct morphological stages (Stages I–VIII) taking place over the course of 8 days [28, 29]. Consequently, our subsequent examination involved an investigation into the potential impact of *BcWRKY33A* overexpression on lateral root initiation in Bok choy. Notably, our findings revealed that the *35S:BcWRKY33A-GFP* transgenic roots displayed significantly fewer lateral root primordia at Stage I compared to the *35S:GFP* roots. However, there was no substantial variation in the number of lateral root primordia observed at Stages II–V (morphogenetic stages; Fig. 2g). Interestingly, at Stages VI–VIII, the *35S:BcWRKY33A-GFP* roots exhibited a significantly higher number of lateral root primordia compared to the *35S:GFP* roots (Fig. 2g), suggesting that the overexpression of *BcWRKY33A* could contribute to the initiation of lateral roots in Bok choy.

Next, we longitudinally dissected the transgenic roots aged 7–9 days and observed that the lateral root primordia developed at a faster rate, resulting in a significantly larger root apical meristem in the *35S:BcWRKY33A-GFP* roots compared to the *35S:GFP* roots (Fig. 2h). These findings collectively imply that the overexpression of *BcWRKY33A* in Bok choy predominantly stimulates lateral root initiation. Additionally, a higher abundance of root hairs was observed in the 16-day-old *35S:BcWRKY33A-GFP* roots compared to the 16-day-old *35S:GFP* roots (Fig. 2i), indicating that the overexpressed *BcWRKY33A* also induces the formation of root hairs in Bok choy.

**Fig. 3:**
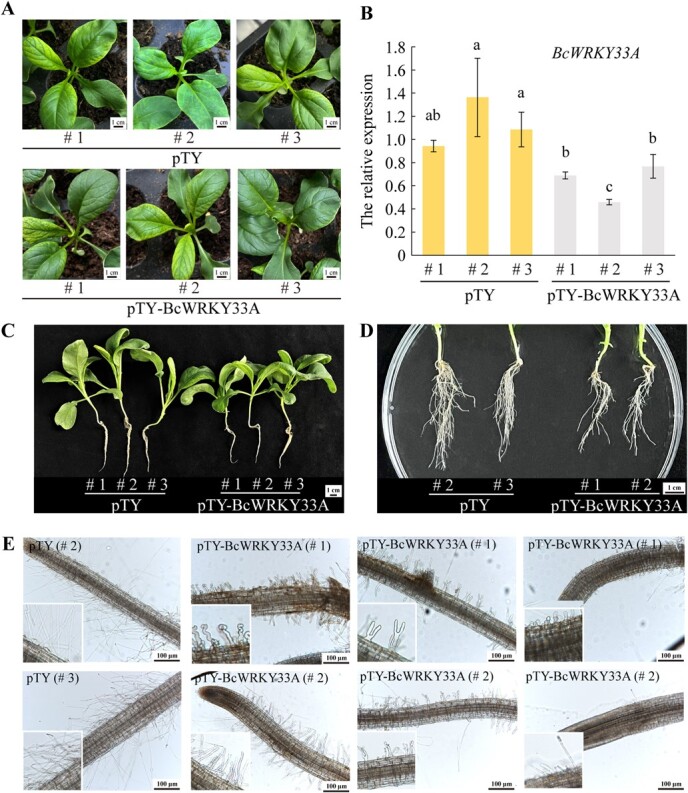
**Silencing of *BcWRKY33A* inhibits root elongation and root hair formation in Bok choy.** (**A–B**) Leaf phenotype (**A**) and *BcWRKY33A* expression levels (**B**) in the control (pTY, #1, #2, #3) and *BcWRKY33A*-slienced (pTY-BcWRKY33A, #1, #2, #3) plants. pTY, the negative control plants. Error bars represent SD. The data are the mean ± SD of three biological replicates and three technical replicates. Different letters indicate statistically significant differences at the level of *P* < .05 (Student’s *t*-test). **(C–E)** The seedlings (**C**), roots (**D**), and root hairs (**E**) phenotypes of *BcWRKY33A*-slienced (pTY-BcWRKY33A) and control (pTY) plants.

### Silencing *BcWRKY33A* inhibits both root elongation and root hair formation in Bok choy.

To get a deeper comprehension of BcWRKY33A’s function in promoting root elongation and root hair development, we employed Virus-Induced Gene Silencing (VIGS) technique to suppress its expression in Bok choy. Following infection, successfully silenced plants exhibited viral mottled symptoms on leaves and reduced expression of *BcWRKY33A* (Fig. 3a–3b). Phenotypic observations revealed that BcWRKY33A-silenced plants exhibited shorter roots (Fig. 3c) and fewer lateral roots (Fig. 3d) compared to the control plants. Moreover, we noted that the root hairs of BcWRKY33A-silenced plants exhibited significantly inhibited growth, displaying a curling or bifurcating phenotype (Fig. 3e), in contrast to the normal growth pattern of root hairs in control plants. Collectively, these findings indicate that silencing *BcWRKY33A* hinders both primary root elongation and root hair formation in Bok choy.

### DNA affinity purification sequencing of *BcWRKY33A*

**Fig. 4:**
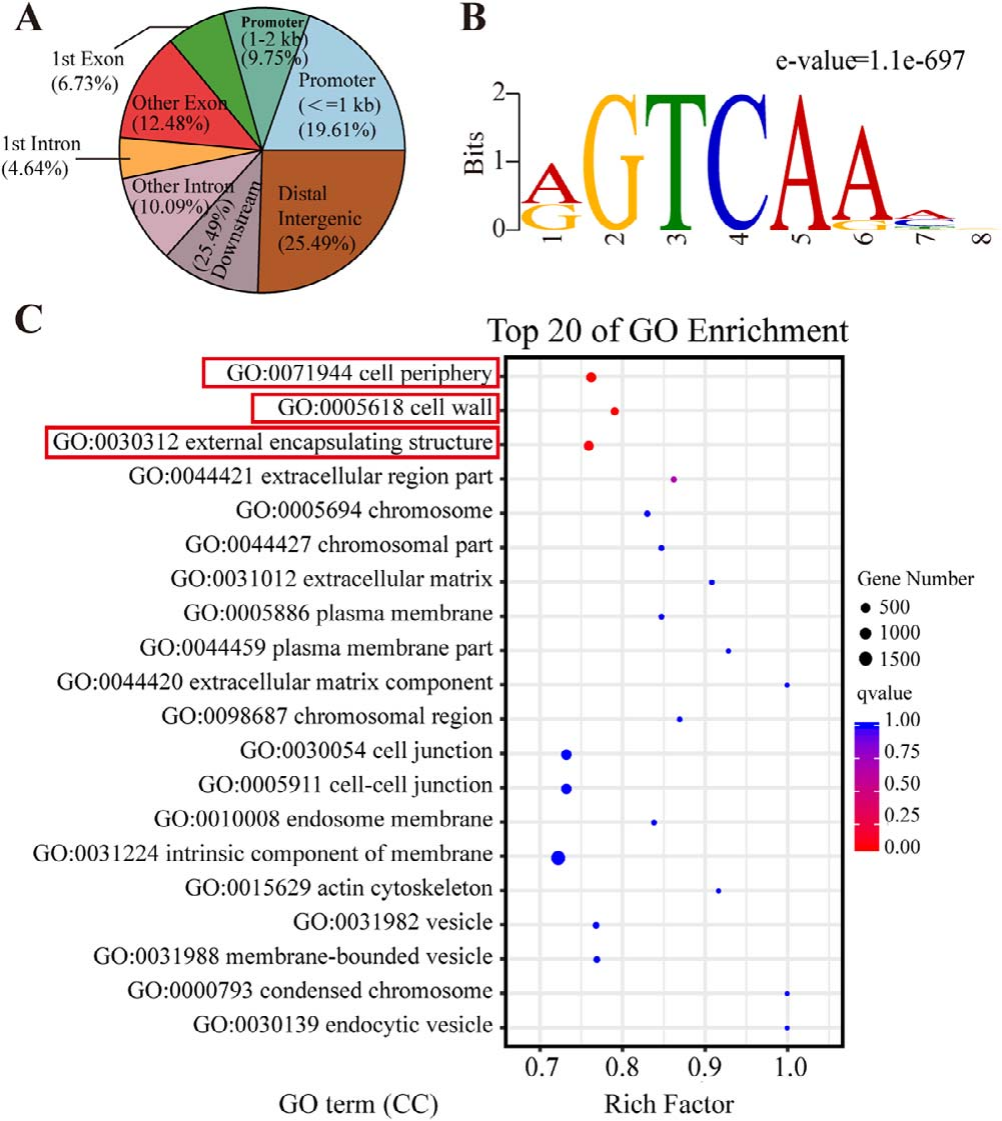
**DAP-seq analysis of the BcWKRY33A protein in Bok choy.** (**A**) Distribution of the BcWRKY33A protein binding regions in Bok choy genomic categories. (**B**) The W-box motif is the top-scoring motif in the BcWRKY33A protein binding regions. (**C**) Top 20 of GO enrichment map of DAP-seq data involved in cellular component (CC).

In order to further investigate the downstream signaling pathway of *BcWRKY33A* in regulating root development, DNA affinity purification sequencing (DAP-seq) was conducted using BcWRKY33A-Halo recombinant protein in Bok choy. While the reads were analyzed from the 2-kb upstream of the transcription start sites (TSSs) to the 2-kb downstream of the transcription termination sites (TTSs), they exhibited clear enrichment around the TSSs (Fig. S3). Out of the 67 550 binding peaks, 29.36% were located within the 2000-bp promoter regions of the target genes. Additionally, ~19.21% and 14.73% of the peaks were identified in exons and introns (Fig. 4a), respectively, while none were detected in untranslated regions (UTRs). Upon analyzing the promoter sequences with the MEME suite to detect the potential binding sites of *BcWRKY33A*, it was revealed that the W-box (G/AGTCAAA/G) was the most enriched cis-element among BcWRKY33A’s binding sites, with a maximum motif length of 8 nt (Fig. 4b). This finding is consistent with previous reports [30, 31].

Subsequently, Gene Ontology (GO) term enrichment analysis was conducted to investigate the relevance of the identified BcWRKY33A-target genes. The results indicated that the peaks in our dataset were predominantly linked to the molecular functions GO terms ‘nucleic acid binding transcription factor activity’, ‘hydrolase activity’, and ‘protein kinase activity’ (Fig. S4a), as well as the biological process GO terms ‘response to external stimulus’, ‘response to external biotic stimulus’, and ‘response to other organism’ (Fig. S4b), implying that *BcWRKY33A* might be responsive to a diverse range of external stimuli. Interestingly, the sets of target genes were significantly linked with cellular component GO terms ‘cell periphery’, ‘cell wall’, and ‘external encapsulating structure’, suggesting that *BcWRKY33A* might have a crucial function in cell organization (Fig. 4c).

Subsequently, our main focus was on the target genes related to root development, encompassing biological processes such as primary root development (GO:0080022), lateral root morphogenesis (GO:0010102), lateral root development (GO:0048527), lateral root formation (GO:0010311), root hair elongation (GO:0048767), root hair cell differentiation (GO:0048765), and root hair cell tip growth (GO:004876). Finally, we identified twelve candidate target genes (Table S1), and further investigation focused on nine of them (*BcATL42–1*, *BcATL42–2*, *BcATL42–3*, *BcPRP3–1*, *BcPRP3–2*, *BcIAA14*, *BcCOW1*, *BcLRP1*, and *BcPKL*) which possessed the W-box in their promoter regions (as indicated in the ‘annotation’ column of Table S1).

### BcWRKY33A directly binds to the W-boxes in the *BcCOW1* and *BcLRP1* promoters

**Fig. 5:**
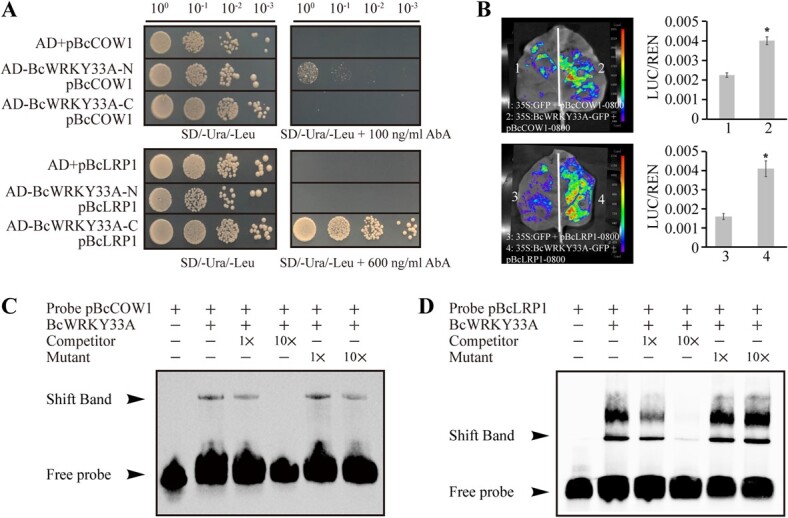
**BcWRKY33A directly binds to the W-box in the promoters of *BcCOW1* and *BcLRP1.*** (**A**) Y1H showed that BcWRKY33A bound to the promoters of *BcCOW1* and *BcLRP1*. (**B**) LUC reporter assay showed that BcWRKY33A activates the expression of *BcCOW1* and *BcLRP1*. (**C-D**) EMSA showed that BcWRKY33A bound to the W-box in the promoter of *BcCOW1* (**C**) and *BcLRP1* (**D**). The experiments were performed twice with the same results. Error bars represent SD. The data are the mean ± SD of three biological replicates. ^*^P < 0.05 (Student’s *t*-test).

To investigate the direct binding of BcWRKY33A to the promoters of the nine candidate target genes, a yeast one-hybrid (Y1H) assay was conducted. Following our previous study [26], the BcWRKY33A protein was truncated into nBcWRKY33A (1–158 amino acids (aa)) and cBcWRKY33A (159–476 aa) to mitigate the toxic effects of the full-length BcWRKY33A on yeast. These truncated proteins were individually inserted into the pGADT7 vector. The Y1H assay revealed that nBcWRKY33A bound to the *BcCOW1* promoter and cBcWRKY33A interacted with the *BcLRP1* promoter (Fig. 5a), while neither protein exhibited binding to the promoters of the remaining seven genes (Fig. S5). Additionally, dual-luciferase (LUC) reporter assay conducted in tobacco leaves further validated the binding capability of BcWRKY33A to the promoters of *BcLRP1* and *BcCOW1* (Fig. 5b), indicating that BcWRKY33A can directly bind to the promoters of *BcCOW1* and *BcLRP1*.

Based on the DAP-seq data, which suggested that BcWRKY33A predominantly binds to the W-box, we designed biotin-labeled probes pBcLRP1 and pBcCOW1, respectively, containing the conserved W-box according to the promoter sequences of *BcLRP1* and *BcCOW1* (Table S1). A mutated W-box probe was also designed and applied as a negative control. As expected, the electrophoretic mobility shift assay (EMSA) results confirmed the direct binding of BcWRKY33A to the W-box in the promoters of *BcLRP1* and *BcCOW1* (Fig. 5c; 5d). Collectively, these findings demonstrate that BcWRKY33A can directly interact with the promoters of *BcCOW1* and *BcLRP1*.

### Overexpression of *BcLRP1* and *BcCOW1* promote primary root elongation and root hair development respectively in Bok choy

**Fig. 6:**
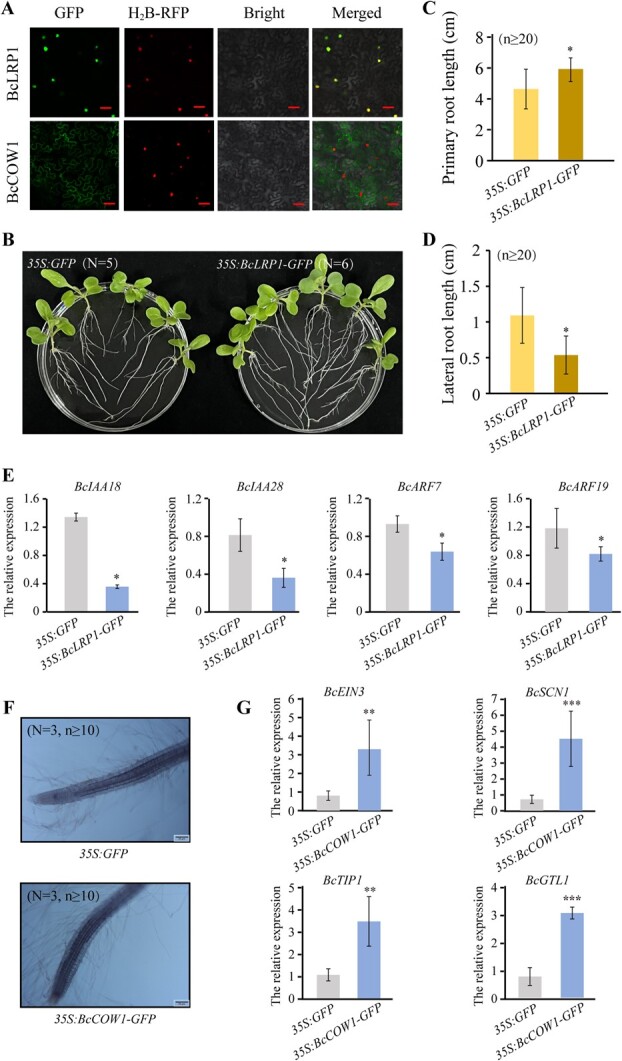
**Overexpression of *BcLRP1* and *BcCOW1* promote primary root elongation and root hair development, respectively, in Bok choy.** (**A**) Subcellular localization of BcLRP1 and BcCOW1 proteins in tobacco leaves. H_2_B-RFP was used as a nuclear marker. (**B – D**) The root phenotype (**B**), the primary root length (**C**), and lateral root length (**D**) of *35S:GFP* and *35S:BcLRP1-GFP* Bok choy. At least five seedlings per line were used. *N* represents number of seedlings, *n* represents number of roots. (**E**) The relative expression levels of genes involved in lateral root development in *35S:GFP* and *35S:BcLRP1-GFP* Bok choy. (**F**) The root hair phenotype of *35S:GFP* and *35S:BcCOW1-GFP* Bok choy. *N* represents number of seedlings, three seedlings per line were used, *n* represents number of roots. (**G**) The relative expression levels of genes involved in root hair development in *35S:GFP* and *35S:BcCOW1-GFP* Bok choy. Error bars represent SD. The data are the mean ± SD of three biological replicates. ^*^P < 0.05, ^**^P < 0.01, ^***^P < 0.001 (Student’s *t*-test).

In *Arabidopsis*, *LRP1* has been identified as an auxin-inducible gene expressed at all stages of lateral root development and *COW1* has been described as involved in root hair morphology [32, 33]. To understand the functions of *BcLRP1* and *BcCOW1* in Bok choy, their expression was first detected in different tissues of Bok choy using qRT-PCR. *BcLRP1* was mainly expressed in root, stem, and shoot, while *BcCOW1* was mainly expressed in flower and stem (Fig. S6a; S6b). Interestingly, after a 24-h treatment with 150 mM NaCl, the expression of both *BcLRP1* and *BcCOW1* in the root increased significantly (Fig. S6a; S6b), while the expression of *BcLRP1* in the leaf, petiole, and shoot and that of *BcCOW1* in the flower decreased significantly. Analysis of subcellular localization suggested that the BcLRP1 protein localized in the nucleus, while the BcCOW1 protein localized in the plasma membrane (Fig. 6a).

Using *A. rhizogenes*-mediated transformation [27], we generated *35S:BcLRP1-GFP* and *35S:BcCOW1-GFP* transgenic roots in Bok choy (Fig. S6c; S6d). We found that *35S:BcLRP1-GFP* transgenic roots had significantly longer primary roots but shorter lateral roots compared with *35S:GFP* (Fig. 6b–6d). Since auxin-responsive genes *IAA18*, *IAA28*, *ARF7*, and *ARF19* have been reported to promote lateral root development [7, 8], we detected the expression level of them in *35S:BcLRP1-GFP* Bok choy. As expect, *IAA18*, *IAA28*, *ARF7*, and *ARF19* was significantly reduced in *35S:BcLRP1-GFP* roots compared to *35S:GFP* (Fig. 6e), suggesting that enhancing the expression of *BcLRP1* in Bok choy roots leads to an augmentation of primary root length while concurrently suppressing the formation of lateral roots.

In contrast, the *35S:BcCOW1-GFP* transgenic roots showed increased root hair abundance compared to *35S:GFP* (Fig. 6f). Previous studies have demonstrated the importance of Arabidopsis *SCN1*, *EIN3*, *TIP1*, and *GTL1* in root hair growth [9–12]. Then our results revealed a significant upregulation of *BcEIN3*, *BcSCN1*, *BcTIP1*, and *BcGTL1* in *35S:BcCOW1-GFP* transgenic roots compared to the control (Fig. 6g), indicating that elevating *BcCOW1* expression in the transgenic Bok choy roots positively influences root hair development.

### BcWRKY25 is induced by salt stress and acts as an upstream regulator of *BcWRKY33A*

**Fig. 7:**
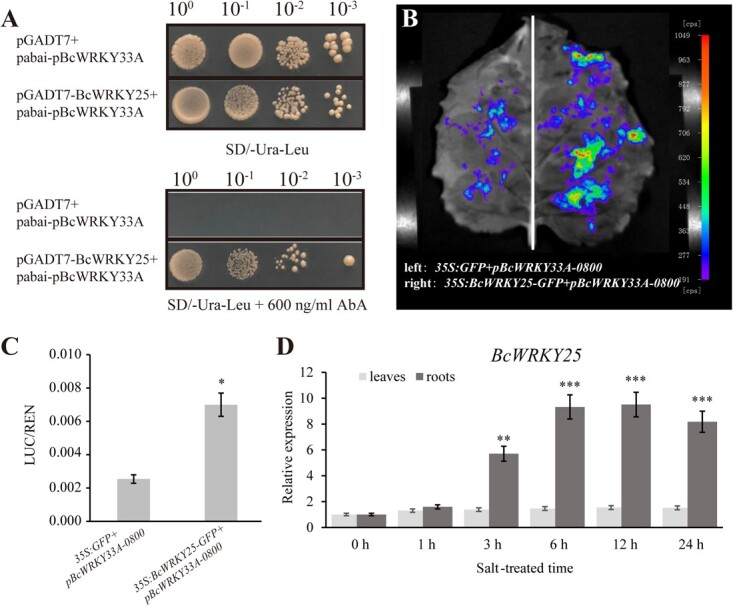
***BcWRKY25* directly binds to activate *BcWRKY33A* expression.** (**A**) BcWRKY25 binds to the p*BcWRKY33A* in the Y1H system. (**B**) Representative LUC luminescence image and (**C**) LUC/REN ratio on tobacco leaves co-infiltrated with the *Agrobacterium* strains containing *35S:BcWRKY25* and p*BcWRKY33A:LUC* (Right)*. Agrobacterium* strains containing *35S:GFP* and p*BcWRKY33A:LUC* were used as the negative control (Left). (**D**) The relative expression of *BcWRKY25* in leaves and roots of salt-treated NHCC ‘Suzhouqing’ plants for 24 h. Error bars represent SD. The data are the mean ± SD of three biological replicates. ^**^P < 0.01, ^***^P < 0.001 (Student’s *t*-test).

*BcWRKY33A* can affect root development and regulate plant salt tolerance (Figs. 2 and 3) [23]. To further explore its regulatory network, we conducted a Y1H assay using the *BcWRKY33A* promoter (p*BcWRKY33A*) as bait to identify the transcription factors that regulate its expression. We identified 24 proteins, and three of them (*BraC03g016460.1*, *BraC03g045330.1*, and *BraC03g041750.1*) were found to be involved in plant salt tolerance (Table S2). *BraC03g045330.1* is a homolog of Arabidopsis *WRKY25* in Bok choy. Given that previous research has shown that *WRKY25* is transcriptionally regulated during early salt stress in 24 h [34], and overexpression of either *WRKY25* or *WRKY33* can enhance NaCl tolerance in Arabidopsis [35], we further confirmed the direct interaction between BcWRKY25 and the *BcWRKY33A* promoter using Y1H system and LUC reporter assay (Fig. 7a–c). Next, we further verified the expression pattern of *BcWRKY25* under salt stress, and the results showed that its expression was significantly upregulated in roots under salt treatment with the extension of treatment time, indicating that *BcWRKY25* directly responded to salt stress (Fig. 7d).

## Discussion

Plant roots play multiple important roles, including absorbing water and nutrients, providing stability, and interacting with soil organisms. To adapt to changing environments, genes involved in root development need to be constantly regulated [5, 36]. The WRKY family, a prominent group of transcription factors, is known to be involved in plant growth and development [13]. Specifically, *WRKY33* has been shown to be upregulated under salt stress, and its mutation leads to inhibited root growth [34]. For instance, WRKY33 induces *AtCYP94B1* to promote the formation of root apoplastic barrier and enhancing salt tolerance [21]. Additionally, *WRKY33* could act as a negative regulator in response to phosphate deficiency, influencing the architecture of the root system [22]. In our previous study, the upregulation of *BcWRKY33A* has been shown to enhance the viability and root elongation of transgenic *Arabidopsis* plants exposed to saline conditions. Conversely, the silencing of *BcWRKY33A* expression in Bok choy has been observed to induce symptoms of wilting and yellowing, accompanied by diminished photosynthetic efficiency and elevated levels of reactive oxygen species when subjected to salt stress [23]. These findings highlight the significance of *WRKY33* in regulating root development under salt stress [34].

Herein, we discovered that BcWRKY33A directly binds to the promoters of *BcCOW1* and *BcLRP1* through W-boxes (Figs. 4a; 4b; 5). Previous research has shown that *AtLRP1*, an auxin-inducible gene, is expressed throughout various stages of root development and its overexpression in Arabidopsis leads to elongated primary roots and reduced lateral root density [32]. Similarly, we observed that overexpressing *BcLRP1* in Bok choy promotes primary root elongation while inhibiting lateral root formation (Fig. 6b–6d). Interestingly, as an upstream regulator of *BcLRP1*, overexpressing *BcWRKY33A* also resulted in longer primary roots (Fig. 2a–2f), increased lateral root primordia at later Stages I, IV, and V, and induced root hair formation (Fig. 2g; 2i). This suggests that *BcWRKY33A* plays a role in promoting primary root elongation by regulating the expression of *BcLRP1* in Bok choy. However, *BcWRKY33A* may also have a separate mechanism for promoting lateral root development in Bok choy, which does not involve *BcLRP1*.

*COW1* encodes a Sec14p-like phosphatidylinositol transfer protein [33]. Null mutants of *AtCOW1* in Arabidopsis and its homology *OsSNDP1* in rice display shortened and irregularly shaped root hairs [33, 37]. In our study, we discovered that *BcCOW1* is directly activated by BcWRKY33A (Fig. 5) and overexpression of *BcCOW1* enhances root hair development (Fig. 6f). We also observed increased expression of root hair developmental genes *BcSCN1*, *BcEIN3*, and *BcTIP1* in *BcCOW1* overexpression roots of Bok choy (Fig. 6g). These findings align with our observation that overexpression of *BcWRKY33A* leads to abundant and well-developed root hairs (Fig. 2i), while silencing of *BcWRKY33A* results in aberrant root hair phenotypes (Fig. 3e). Therefore, we propose that *BcWRKY33A* promotes root hair development in Bok choy by directly activating *BcCOW1* expression.

Additionally, WRKY25 and WRKY33 frequently occurred together in response to abiotic stress. For example, WRKY25, WRKY26, and WRKY33 can increase transgenic *Arabidopsis* resistance to high temperature stress by altering the *HSP101* and *ZAT10* [38]. ABA and NaCl treatments enhanced the transcriptional abundance of *WRKY25* and *WRKY33*, and overexpression of these genes boosted NaCl tolerance in transgenic *Arabidopsis* [35]. Overexpression of *WRKY25* and *WRKY33* mediated transcriptional control of zinc stress genes *ZIP3* and *ZIP4*, downstream of AGB1, thereby enhancing the tolerance of transgenic *Arabidopsis* to zinc deprivation [39]. Based on our results, we proposed that BcWRKY25 operates on the upstream of *BcWRKY33A*, controls its expression, and participates in the processes of salt resistance and root growth (Fig. 7), which gives a credible explanation for the phenomena that WRKY25 and WRKY33 always jointly resist abiotic stress.

**Fig. 8:**
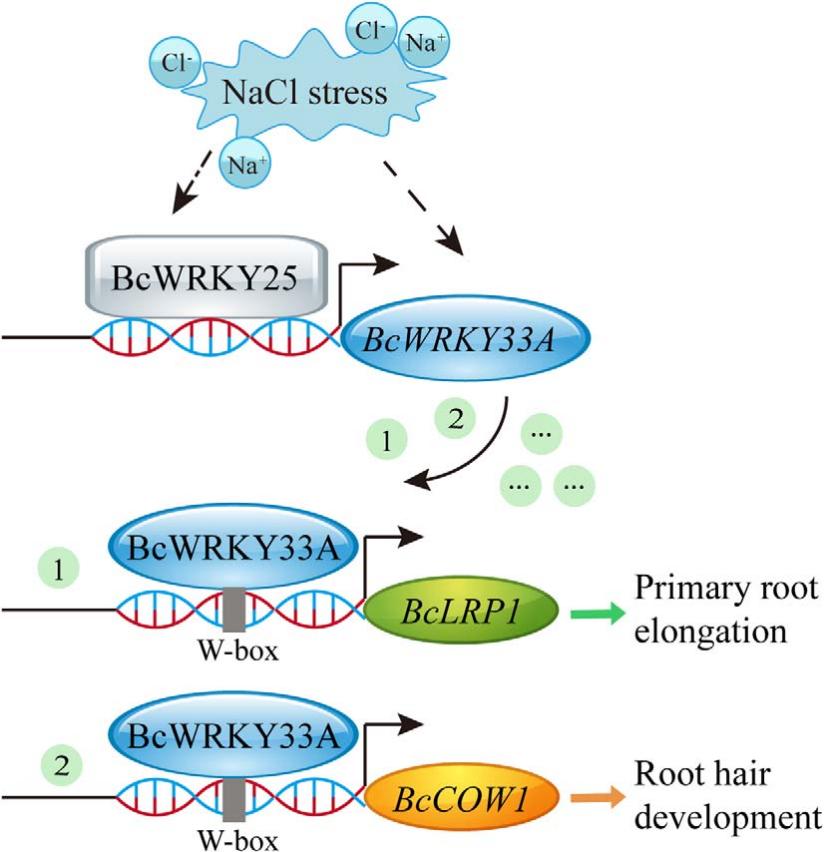
**A proposed working model for *BcWRKY33A*-mediated root development in Bok choy.** The transcription levels of *BcWRKY25* and *BcWRKY33A* increased when exposed to NaCl stress. BcWRKY25 binds to the promoter of *BcWRKY33A*, enhancing its expression. As a result, the elevated BcWRKY33A directly activates the expression of *BcLRP1* and *BcCOW1*, leading to the promotion of primary root elongation and root hair development.

Collectively, we investigated and demonstrated the involvement of the BcWRKY25-BcWRKY33A-BcLRP1/BcCOW1 module in promoting root development for enhanced salt tolerance in Bok choy. To be specific, Bok choy exhibits upregulation of *BcWRKY25* and *BcWKRY33A* in response to NaCl stress. In the meanwhile, BcWRKY25 binds to the promoter of *BcWRKY33A* to control its expression, while BcWRKY33A operates upstream of *BcLRP1* and *BcCOW1* to increase primary root elongation and stabilize the development and morphology of root hairs, thereby improving plant salt tolerance (Fig. 8).

## Materials and methods

### Plant transformation and growth conditions

Bok choy cv. ‘Suzhouqing’ and *Arabidopsis thaliana* ‘Col-0’ were used in the present study. *35S:BcWRKY33A* transgenic *Arabidopsis* (#1 and #3) was obtained in our previous study [23]. To generate *35S:BcWRKY33A* transgenic Bok choy hairy roots, the hypocotyls of 5-day-old Bok choy ‘Suzhouqing’ seedlings were treated with *A. rhizogenes* (K599) carrying the *35S:BcWRKY33A-GFP* vector. The successfully induced hairy roots were selected for analysis, while the plants inoculated with the empty vector (*35S:GFP*) served as the negative control. All plants were grown under long-day conditions (16 h light / 8 h dark) in an artificial climate chamber at 23°C.

### *β*-Glucuronidase staining and propidium iodide staining

The promoter sequence of *BcWRKY33A*, spanning from −2000 to −1 relative to the translational start codon, was cloned and inserted into the binary vector pFast-G04 using the Gateway system [40]. Transgenic Arabidopsis plants expressing *BcWRKY33A:GUS* were generated in a previous study [23, 40]. To investigate the expression patterns of *BcWRKY33A* under abiotic stress, we treated both WT and *BcWRKY33A:GUS* seedlings with heat (43°C, 6 h), cold (4°C, 6 h), or NaC (100 mM, 24 h). Subsequently, GUS staining and microscopy were performed. As for PI staining, 5-day-old WT and *35S:BcWRKY33A* seedlings were incubated with 10 g/ml PI in the dark for 5 min. Then, the PI-stained seedlings were washed with ddH_2_O water and transferred to a slide for confocal imaging using a laser scanning microscope (ZEISS, LSM780, Germany).

### Paraffin section and microscopic observation

Transgenic plant roots (*35S:BcWRKY33A-GFP* and *35S:GFP*) were fixed in an FAA solution overnight. The solution consisted of 75% ethanol, acetic acid, glycerol, 38% formaldehyde, and glycerin in a ratio of 90:5:5:5:5 ml. After dehydration, embedding, and trimming, the samples were sectioned longitudinally to a thickness of 6 μm for aniline blue staining [41]. Three replicates were observed using a light microscope (Olympus D72, Japan).

### DAP-seq

DAP-seq assays were performed as previously described, with slight modifications [42]. In brief, Bioruptor® Plus sonication device was used to fragment genomic DNA (gDNA) of the ‘Suzhouqing’ plants to a size of 100–400 bp in length. The resulting fragmented gDNA was end-repaired and added with a dA-tail using the NEXTflex Rapid DNA-Seg Kit (Cat. 5144–08). Next, the DAP-Seq adaptor was ligated to the fragmented gDNA with the help of a ligase enzyme. The vector pHalo-Tag was obtained from Gene Denovo Biotech Co., Ltd. (Guangzhou, China). The full-length of *BcWRKY33A* cDNA was inserted into the *EcoR* V-*Hind* III-cut pHalo-Tag vector to generate recombined plasmid pHalo-Tag-BcWRKY33A. The DAP-seq was carried and analyzed by Gene Denovo Biotech Co., Ltd. (Guangzhou, China). Reads were mapped to the *Brassica campestris* genome (http://tbir.njau.edu.cn/NhCCDbHubs/index.jsp) using Bowtie2 [43], and reads depths from 2 k upstream of the TSS to 2 k downstream of TTS were analyzed using deepTools software [44]. Peak calling was performed using Macs2 (*q*-value <.05) [45] and annotation of peak-related genes was conducted using the ChIPseeker R package [46]. Motif discovery was performed using the MEME suite (http://meme-suite.org/).

### Virus-induced gene silencing

In the VIGS system, pTY vector, derived from turnip yellow mosaic virus (TYMV), which is a spherical plant virus with a positive-strand RNA genome, and can infect many members of Brassicaceae [47]. The Bok choy *BcWRKY33A*-silencing lines were generated using the TYMV-based VIGS system. The interfering 40-bp fragment (5’-ATGAATGGTTC TGTTAATTGGTCACAACAAACCGCAAGAGCTCTTGCGGTTTGTTGTGACCAATTAACAGAACCATTCAT-3′) of the *BcWRKY33A* cDNA sequence (underlined) and its antisense sequence were inserted into the pTY vector. A gene gun (Biolistic PDS-1000/He, Bio-Rad) was used to bombard seedlings with the empty or constructed vector [48]. After infection, successfully silencing plants always show viral mottled symptoms on leaves. New leaves from possible successfully silencing plants were collected at 10 days after bombardment to confirm the expression of *BcWRKY33A*. Following quantitative real-time PCR (qPCR), three independent biological replicates and three technical replicates were taken for each line, then we chose *BcWRKY33A*-silenced lines with significant repression of *BcWRKY33A* transcript levels for further experiments.

### Yeast-one-hybrid assay

The 2-kb promoters of nine potential target genes (*BcATL42–1*, *BcATL42–2*, *BcATL42–3*, *BcPRP3–1*, *BcPRP3–2*, *BcIAA14*, *BcCOW1*, *BcLRP1*, and *BcPKL*) involved in root development were amplified from the genomic DNA of ‘Suzhouqing’ and then introduced into the pAbAi vector. The interaction of each promoter with cBcWRKY33A or nBcWRKY33A was then analyzed in Y1H Gold strains with appropriate aureobasidin A (AbA) concentrations.

Additionally, the 2-kb promoter of *BcWRKY33A* was inserted into the pAbAi vector for Y1H library screening, and the recombinant vector pAbAi-pBcWRKY33A was digested with the restriction enzyme *BstB*I. Using Matchmaker Insert Check PCR Mix 1 (Cat. No. 630496, Takara, Japan), the bait yeast strain was verified after construction, and the expression of AbA^r^ was also examined. Then the ‘Suzhouqing’ library was used as prey and cloned into the vector pGADT7. Screening of the Y1H library was performed according to the manufacturer’s protocol (Cat. No. 630491, Takara, Japan).

### Dual-luciferase reporter assay

The 2-kb promoters of *BcLRP1* and *BcCOW1* were cloned individually into the pGreenII-0800-LUC vector as reporters, and *35S:GFP* (control) and *35S:BcWRKY33A-GFP* constructs were used as effectors. After being transformed into *Agrobacterium tumefaciens* GV3101 (pSoup), the mixture of effector and reporter (1:1) was injected into 1-month-old tobacco leaves. After 3 days, D-luciferin was sprayed onto the injected leaves, and the LUC imaging was captured using a plant imaging system (Berthold, Night Shade LB 985). A Dual-Luciferase Reporter Assay Kit was used to detect and measure the ratio of LUC and REN.

### EMSA

The recombinant vector pGEX-BcWRKY33A was transferred into *Escherichia coli* strain BL21 (DE3), which was induced with 1 mM isopropyl *β*-D-thioacetamide (IPTG) at 28°C for 12 h. The BcWRKY33A-GST protein was purified by GST-sefinose resin 4FF (Settled Resin; Biotech Biotech, China). Subsequently, EMSA was performed using a Chemiluminescent EMSA Kit (GS009, Beyotime, China). Purified proteins and biotin-labeled probes were mixed and incubated at room temperature, and bound and unbound DNA–protein complexes were separated by 6% native polypropylene gel electrophoresis and transformed onto nylon membranes (Biosharp, China) with TGE buffer (R23174, Yuanye, China), followed by UV cross-linking and chemiluminescence instrumentation (ChemiDoc MP, USA). All probe sequences were shown in Table S3.

### Subcellular localization analysis

The cDNA sequences of genes *BcCOW1* and *BcLRP1* without the termination codons were fused with a green fluorescent protein (GFP) gene tag individually in the pEarlygate103 vector via the Gateway system. The resulting vectors were transformed into GV3101 and injected into 1-month-old tobacco leaves [49]. The fluorescent signals in tobacco leaves were examined and photographed 96 h post-treatment (hpt) using a laser scanning microscope (ZEISS, LSM780, Germany).

### Quantitative real-time PCR

Total RNA was extracted from various samples using a TRIzaol reagent, and cDNA was synthesized using an Evo M-MLV Mix Kit with gDNA Clean for qPCR (Accurate Biotechnology (Hunan) Co., Ltd., China). All genes’ relative expressions were determined first using the QuantStudio®5 system and Hieff® qPCR SYBR Green Master Mix (Low Rox Plus) (Cat No.11202ES08; Yeasen, Shanghai, China), and then analyzed using the 2^-ΔΔCT^ method as described in [50] and [51]. *ELF4A* (AT1G80000) and *BcGAPDH* (BraC09g068080.1) were used as the internal control genes for *Arabidopsis* and ‘Suzhouqing’, respectively. Table S3 lists all primers used in this study. In our qPCR experiments, we used three technical repeats per sample and at least three biological repeats.

## Supplementary Material

Web_Material_uhae280

## Data Availability

All data generated or analyzed during this study are included in this published article and supplementary files online.
