# Supplementary material for: BcWRKY25-BcWRKY33A-BcLRP1/BcCOW1 module promotes root development for improved salt tolerance in Bok choy
Source: Hortic Res. 2024 Sep 28;12(1):uhae280. doi: 10.1093/hr/uhae280 (PMC11756305; doi:10.1093/hr/uhae280)
Supplement: Web_Material_uhae280 [file web_material_uhae280.zip › Supplementary Figures.pdf]

SUPPLEMENTARY FIGURES

Fig. S1. The effects of overexpressed BcWRKY33A in transgenic *Arabidopsis* on root elongation and root hair formation.

Fig. S2. GFP imaging in *35S:BcWRKY33A-GFP* transgenic Bok choy roots.

Fig. S3. The DAP-seq analysis of the BcWKRY33A protein in Bok choy.

Fig. S4. Top 20 of GO enrichment map of DAP-seq data.

Fig. S5. Y1H assay to verify whether BcWRKY33A directly binds to the promoters of *BcIAA14, BcPRP3-1, BcPRP3-2, BcATL41-1, BcATL42-2, BcATL42-3* and *BcPKL*.

Fig. S6. The expression patterns of *BcLRP1* and *BcCOW1* in Bok choy.

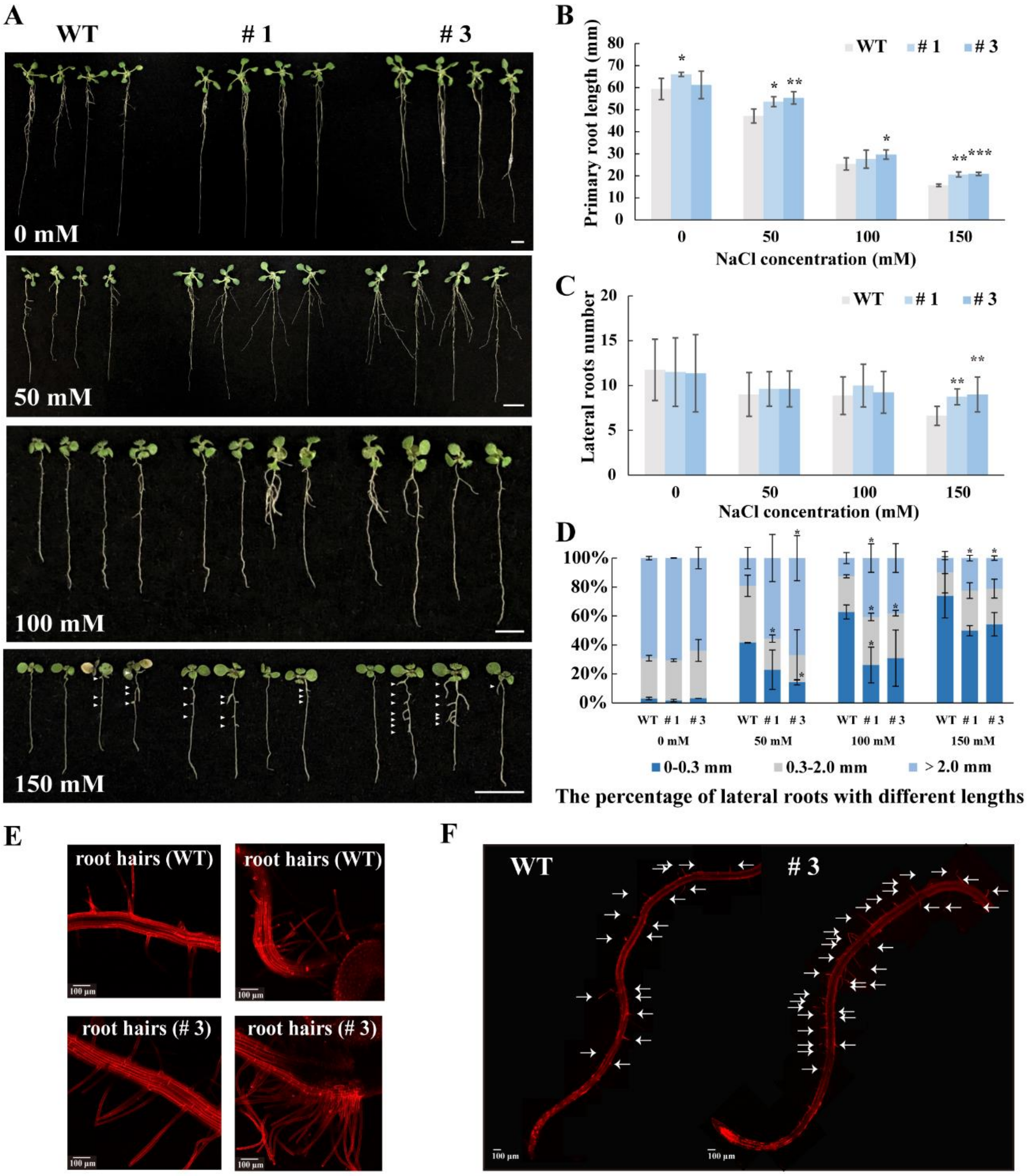

Fig. S1. The effects of overexpressed *BcWRKY33A* in transgenic *Arabidopsis* on root elongation and root hair formation.

(**A–D**) The phenotype (**A**), primary root length (**B**), lateral roots number (**C**), and percentage of lateral root with different lengths (**D**) of *35S:BcWRKY33A* transgenic *Arabidopsis* lines (# 1, # 3) under NaCl treatments. Bars = 1 cm. The white triangles indicate lateral roots in A. (**E–F**) The root hairs (**E**) and the distribution of root hairs (white arrows) on the primary root (**F**) of *35S:BcWRKY33A* transgenic *Arabidopsis* lines (# 3) under the normal growth conditions. Error bars represent SD. The data are the mean ± SD of three biological replicates. $^{*}p < 0.05$, $^{**}p < 0.01$, $^{***}p < 0.001$ (Student's *t*-test).

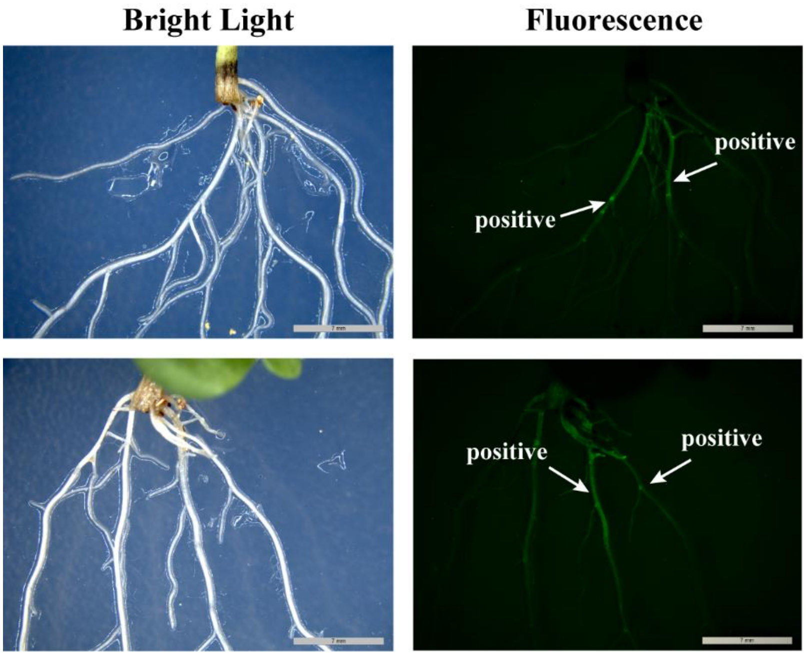

Fig. S2. GFP imaging in *35S:BcWRKY33A-GFP* transgenic Bok choy roots.

Bars = 7 mm.

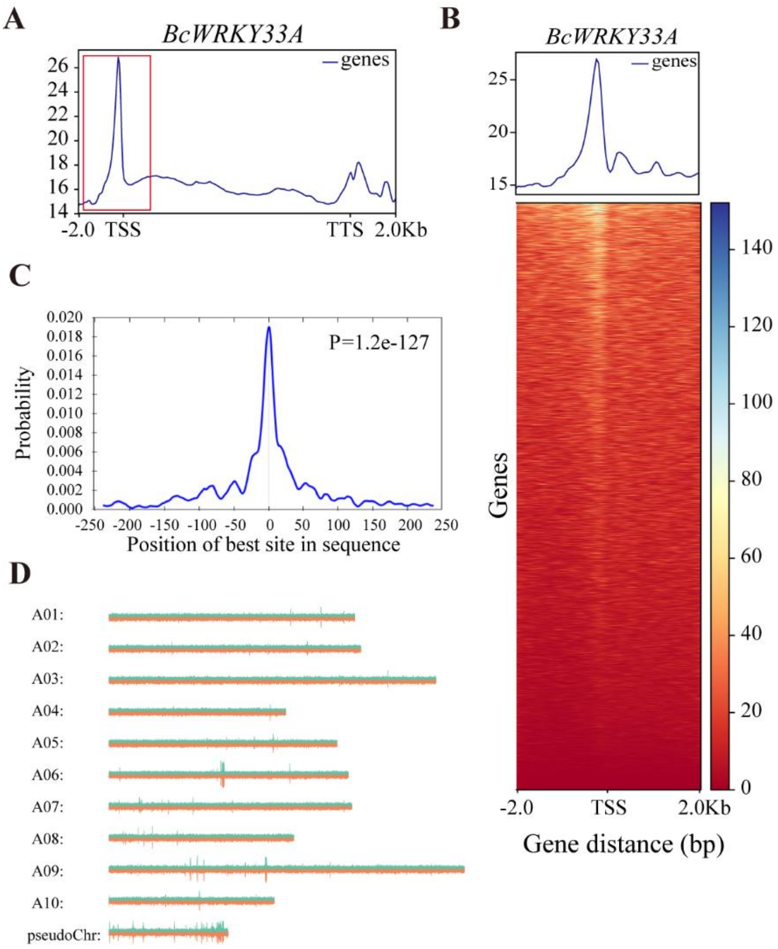

Fig. S3. The DAP-seq analysis of the BcWKRY33A protein in Bok choy.

(**A, B**) The genome sequencing depth distribution. The y axis shows the average sequencing depth of the matched sequence at the specific gene position, and the x axis shows the location of the gene. TTS, transcription termination site. (**C**) Motif position probability for BcWKRY33A at given positions. The x axis indicates the position of the W-box binding motif in the sequence, and the y axis indicates the probability of the identified motif. The significance for central enrichment is described by the *P* value. (**D**) The distribution of reads on chromosomes. The density of reads compared to individual chromosomes on the genome (positive and negative strands) was counted to see the relationship between chromosome length and the number of reads on the comparison.

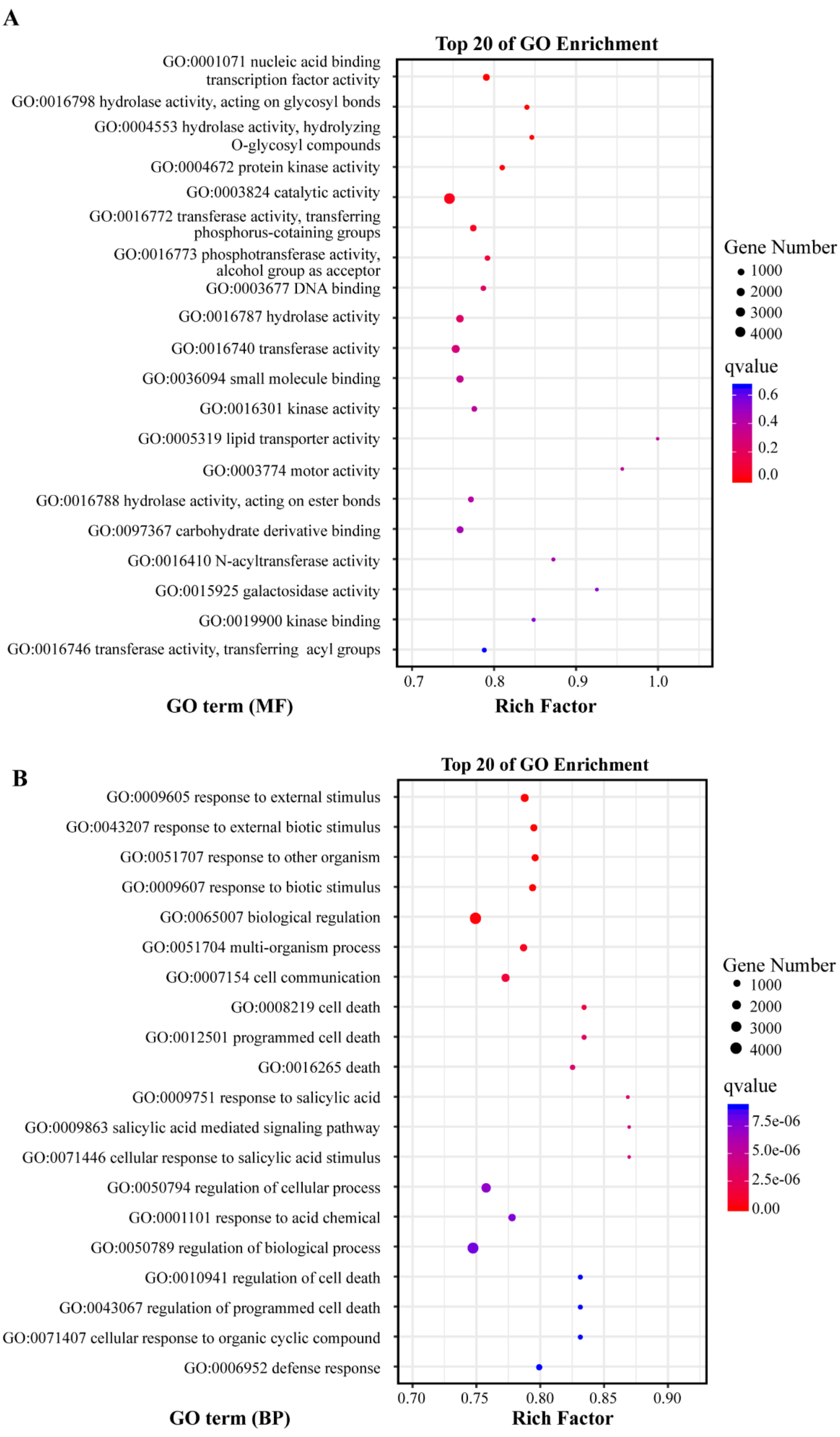

Fig. S4. Top 20 of GO enrichment map of DAP-seq data.

(A) Molecular function. (B) Biological process.

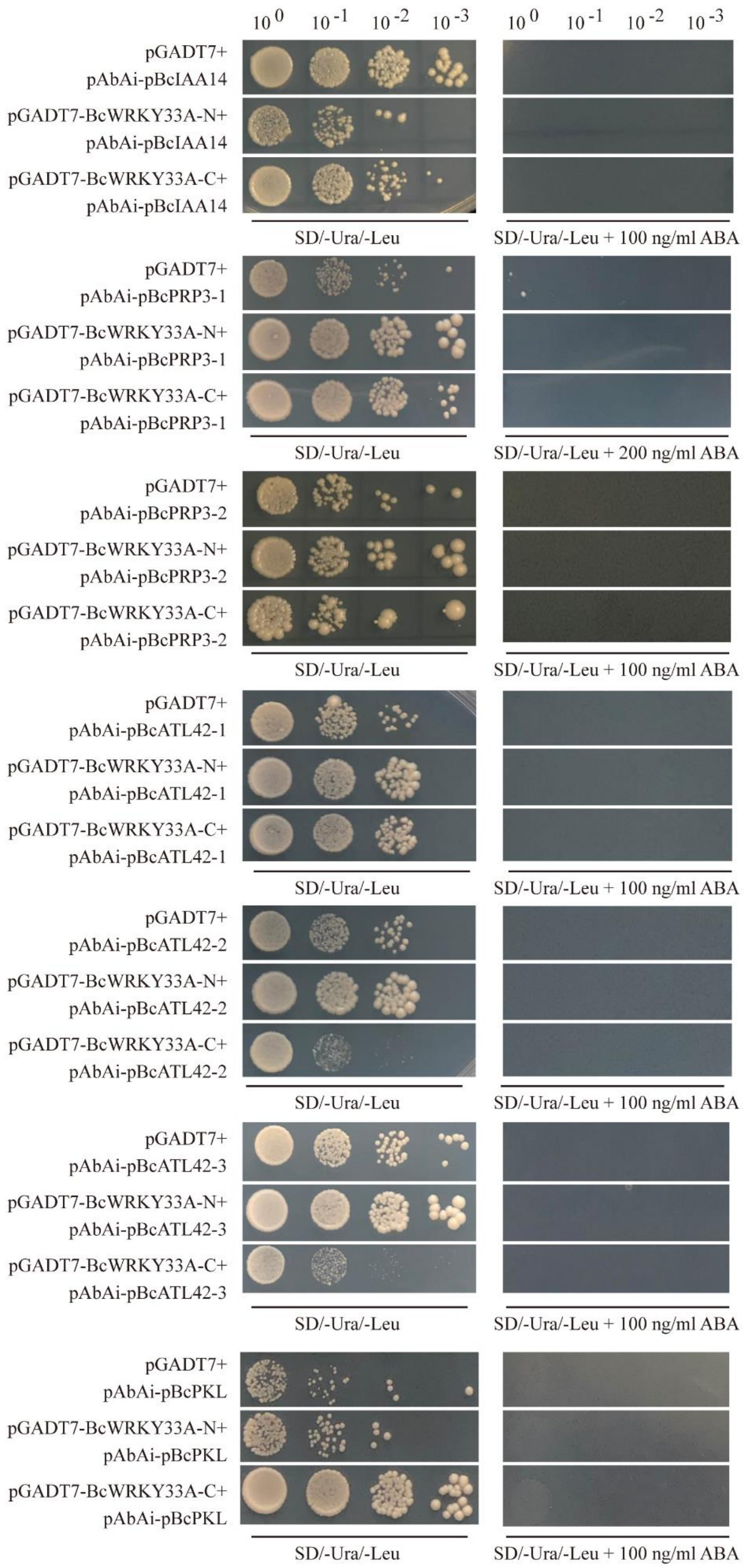

Fig. S5. Y1H assay to verify whether BcWRKY33A directly binds to the promoters of *BcIAA14, BcPRP3-1, BcPRP3-2, BcATL41-1, BcATL42-2, BcATL42-3* and *BcPKL*.

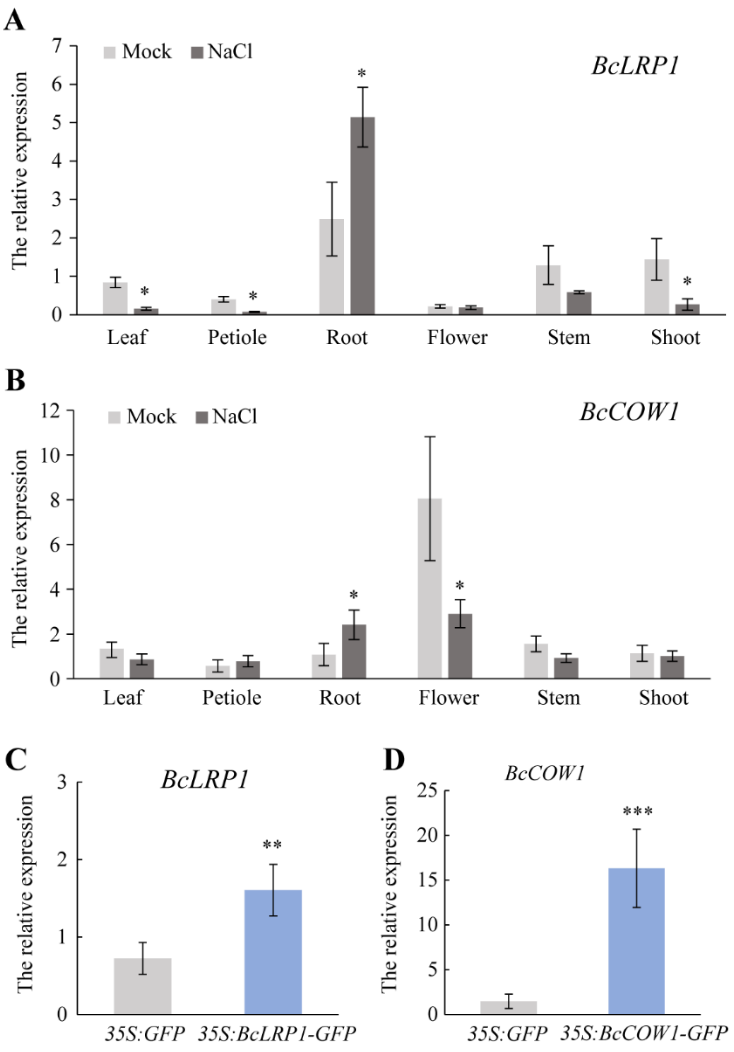

Fig. S6. The expression patterns of *BcLRP1* and *BcCOW1* in Bok choy.

(**A, B**) The relative expression level of *BcLRP1* (**A**) and *BcCOW1* (**B**) in different tissue of one-month-old ‘suzhouqing’ under mock or 150 mM NaCl treatment at 24-hour post treatment (hpt). (**C**) The relative expression levels of *35S:BcLRP1-GFP* transgenic ‘suzhouqing’ hairy roots. (**D**) The relative expression levels of *BcCOW1 35S:BcCOW1-GFP* transgenic ‘suzhouqing’ hairy roots. Error bars represent SD. The data are the mean ± SD of three biological replicates. $^{*}p < 0.05$, $^{**}p < 0.01$, $^{***}p < 0.001$ (Student’s *t*-test).
